# Cancer-associated fibroblasts secreted miR-103a-3p suppresses apoptosis and promotes cisplatin resistance in non-small cell lung cancer

**DOI:** 10.18632/aging.103556

**Published:** 2021-05-17

**Authors:** Haifeng Wang, Haibo Huang, Lijiang Wang, Yan Liu, Ming Wang, Shasha Zhao, Guangjian Lu, Xiaohong Kang

**Affiliations:** 1 Third Ward of Oncology Department, The First Affiliated Hospital of Xinxiang Medical University, Weihui 453100, Henan, China; 2 Respiratory Ward 2, The First Affiliated Hospital of Xinxiang Medical University, Weihui 453100, Henan, China; 3 Respiratory Intensive Care Unit, The First Affiliated Hospital of Xinxiang Medical University, Weihui 453100, Henan, China; 4 Clinical Laboratory, The First Affiliated Hospital of Xinxiang Medical University, Weihui 453100, Henan, China; 5 First Ward of Oncology Department, The First Affiliated Hospital of Xinxiang Medical University, Weihui 453100, Henan, China

**Keywords:** NSCLC, exosome, CAF, miR-103a-3p, cisplatin resistance

## Abstract

Background: The cisplatin resistance of non-small cell lung cancer (NSCLC) patients results in low response rate and overall survival rate. Exosomes contribute to pathological processes of multiple cancers.

Objective: In this study, we explored the function and mechanisms of exosomal miR-103a-3p derived from cancer-associated fibroblast (CAF) in cisplatin resistance in NSCLC.

Results: MiR-103a-3p was highly expressed in CAFs and CAF exosomes, and exosomal miR-103a-3p derived from CAFs in NSCLC. CAFs exosomes co-cultured with NSCLC cells promoted miR-103a-3p expression both in NSCLC cells and its exosomes. Functional experiments showed that exo-miR-103a-3p derived from CAFs promoted cisplatin resistance and inhibited apoptosis in NSCLC cells. Pumilio2 (Pum2) bound with miR-103a-3p in cytoplasm and nucleus, and facilitated packaging into CAF-derived exosomes in NSCLC cells. Further analysis showed Bak1 was a direct target of miR-103a-3p, and miR-103a-3p accelerated cisplatin resistance in NSCLC cells via Bak1 downregulation. *In vivo* tumorigenesis assay showed CAF-derived exosomal miR-103a-3p enhanced cisplatin resistance and inhibited cell apoptosis in NSCLC.

Conclusion: Our study revealed that CAFs-derived exosomal miR-103a-3p promoted cisplatin resistance by suppressing apoptosis via targeting Bak1, which provided a potential therapeutic target for cisplatin resistance in NSCLC.

## INTRODUCTION

Lung cancer is one of the main tumors endangering human health [1]. According to the authoritative Chinese cancer epidemiological survey data, the number of people diagnosed with lung cancer in China reached 733000 in 2015, the number of lung cancer deaths was 610000, which ranks first in cancer [2]. Especially, non-small cell lung cancer (NSCLC) is the most common type with high clinical incidence rate [3]. Currently, surgical resection is the first choice for most lung cancer patients. But because of the occult nature of the disease, most patients are in the middle and late stage of tumorigenesis or primary metastasis at the time of diagnosis [4]. For the patients who are not suitable for surgical treatment, cisplatin-based radiotherapy and chemotherapy has become the main means of clinical treatment [4]. However, the resistance of patients to cisplatin results in low response rate and overall survival rate [5]. Therefore, finding an effective method to reverse cisplatin resistance in NSCLC and exploring its related molecular mechanism has become an urgent scientific problem to be solved.

Exosomes are nanoscale vesicles produced by endocytosis, fusion and exudation in various cells, which contain proteins, lipids, genes, coding RNAs, non-coding RNAs and other active biomolecules. Thus, exosomes play an important role in physiological and pathological processes [6]. Exosomes of lung cancer cells released of TGF-β and IL-10 into the tumor microenvironment, which improved the migration ability and promoted metastasis in lung cancer cells [7]. In addition, bone marrow mesenchymal stem cell (BMMSC) derived exosomal miR-144 could inhibit the development of NSCLC, and the potential mechanism might be a downregulation of CCNE1 and CCNE2 [8]. Exosomes can be also secreted by cancer-associated fibroblasts (CAFs), which involves in the process of tumor metastasis and chemoresistance of cancer cells [9, 10]. In breast cancer, CAFs secreted exosomes containing miR-181d-5p into tumor environment, thereby facilitating the EMT process, which possibly modulated through CDX2/HOXA5 axis [11]. In addition, CAFs-derived miR-98-5p promoted the proliferation of ovarian cancer cells, and promoted cisplatin resistance in nude mice [12]. MiR-103a-3p is a highly conserved RNA. Yet, the function of miR-103a-3p in cell-cell communication between CAFs and NSCLC cells and the underlying mechanism remains unclear.

In this study we revealed the novel function of miR-103a-3p in the crosstalk between CAFs and NSCLC cells that promotes cancer progression by inhibiting apoptosis. This study reveals a novel way for prevention and treatment of NSCLC.

## RESULTS

### Exosomal miR-103a-3p derived from CAFs in NSCLC

**Figure 1:**
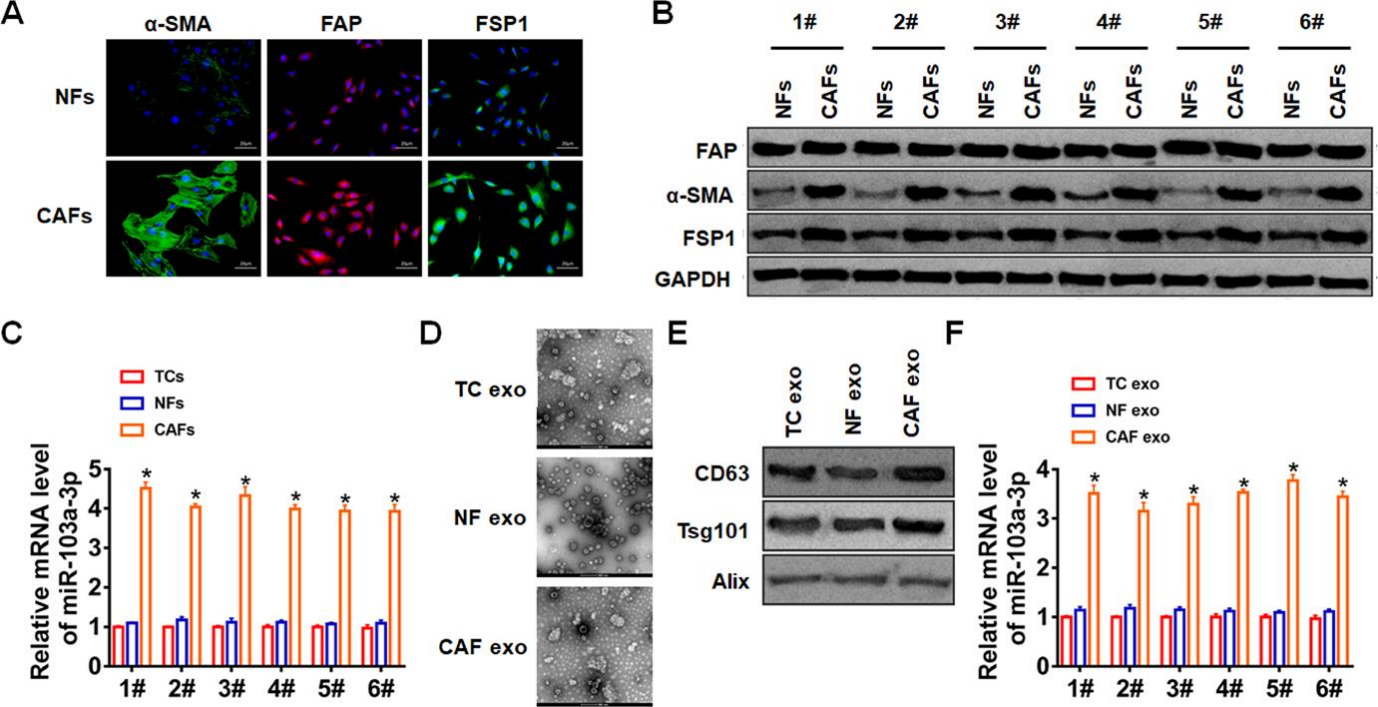
**CAFs secreted exosomal miR-103a-3p in NSCLC.** (**A**) Immunofluorescence staining and (**B**) Western blot for α-SMA, FAP, and FSP1 expression of NFs and CAFs. (**C**) qRT-PCR analyzed the expression of miR-103a-3p in TCs, NFs and CAFs. (**D**) TEM of exosomes isolated from TCs, NFs and CAFs. (**E**) The expression of CD63, Alix, and Tsg101 in exosomes was detected by western blot. (**F**) The expression of miR-103a-3p in exosomes form TCs, NFs and CAFs was tested by qRT-PCR. *p<0.05.

Firstly, we isolated CAFs from NSCLC cancer tissue and normal fibroblasts (NFs) from para-carcinoma tissue. And we found the specific markers for fibroblasts were significantly increased in CAFs (Figure 1A, 1B). Then, we performed qRT-PCR analysis to determine miR-103a-3p level in NFs, tumor cells (TCs) and CAFs. It showed that miR-103a-3p level was the highest in CAFs (Figure 1C). Furthermore, exosomes in NFs, TCs and CAFs were isolated. TEM data showed the morphology of exosomes (Figure 1D), and exosomes markers were detected by western blot (Figure 1E). Interestingly, the expression of miR-103a-3p was the highest in CAF exosomes compared with NF and TC exosomes (Figure 1F). These data indicated exosomal miR-103-3p (exo-miR-103a-3p) derived from CAFs.

### Exo-miR-103a-3p suppressed apoptosis and promoted cisplatin resistance in NSCLC cells

**Figure 2:**
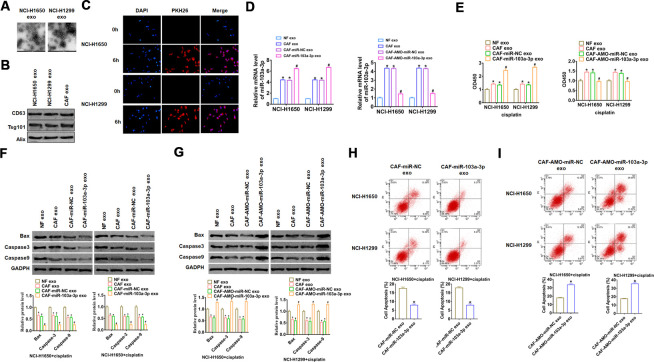
**CAFs secreted exo-miR-103a-3p to suppress apoptosis of NSCLC cells.** (**A**) TEM images of exosomes isolated from NSCLC cell lines NCI-H1650 and NCI-H1299. (**B**) Western blot for CD63, Alix, and Tsg101 in exosomes from NCI-H1650 and NCI-H1299 cells. (**C**) Immunofluorescence staining of PKH-26 labelled CAF exosomes in NCI-H1650 and NCI-H1299 cells. DAPI indicates nucleus. Exosomes from NFs and CAFs transfected miR-103a-3p or AMO-miR-103a-3p or its NC were isolated, then NCI-H1650 and NCI-H1299 cells were incubated with NF or CAF exosomes. (**D**) qRT-PCR analyzed the expression of miR-103a-3p NCI-H1650 and NCI-H1299 cells. (**E**) CCK8 was used to test viability of NCI-H1650 and NCI-H1299 cells. (**F**, **G**) The expressions of apoptosis related protein Bax, Caspase3 and Caspase9 were analyzed by western bolt. (**H**, **I**) The number of apoptotic cells was calculated by flow cytometry. *p<0.05 vs NF exo or, CAF-miR-NC exo or CAF-AMO-miR-NC exo, ^#^ p<0.05 vs CAF-miR-NC exo or CAF-AMO-miR-NC exo.

To verify whether CAF exosomes could enter NSCLC cells, we isolated CAFs exosomes and co-cultured with NSCLC cell lines NCI-H1650 and NCI-H1299. Then the exosomes of NCI-H1650 and NCI-H1299 were isolated and captured by TEM photos (Figure 2A), and exosomes markers were detected by western blot (Figure 2B). As well, we used PKH-26 to label CAF exosomes, and PKH-26 positive exosomes were detected in NCI-H1650 and NCI-H1299 cells after 6 hours of co-culture with CAF exosomes (Figure 2C), which indicated that CAF exosomes entered NCI-H1650 and NCI-H1299 cells and promoted the expression of exo-miR-103a-3p.

To evaluate the role of exo-miR-103a-3p in apoptosis and cisplatin resistance in NSCLC cells, we isolated exosomes from NFs and CAFs transfected miR-103a-3p or AMO-miR-103a-3p or its NC, then NCI-H1650 and NCI-H1299 cells were incubated with NF or CAF exosomes. And we found that miR-103a-3p expression was significantly upregulated upon incubation with exosomes from CAFs with miR-103a-3p overexpression but not with CAFs with AMO-miR-103a-3p (Figure 2D). Functionally, we performed CCK8 assay to estimate cell viability upon cisplatin treatment. It showed that NCI-H1650 and NCI-H1299 cells became insensitive to cisplatin with exosomes from CAFs, especially CAFs transfected with miR-103a-3p (Figure 2E). On the contrary, sensitivity to cisplatin was increased when incubated with exosomes from AMO-miR-103a-3p transfected CAFs. In addition, CAFs exosomes incubation decreased the expression of apoptosis related protein Bax, Caspase-3 and Caspase-9, and exosomes from miR-103a-3p transfected CAFs further inhibited NCI-H1650 and NCI-H1299 apoptosis (Figure 2F), while exosomes from AMO-miR-103a-3p transfected CAFs incubation abolished the anti-apoptotic effect of CAFs exosomes incubation (Figure 2G). The results of cytometry also proved that forced expression of miR-103a-3p inhibited apoptosis upon cisplatin treatment (Figure 2H), while knockdown of miR-103a-3p exhibited a pro-apoptotic role (Figure 2I). Together, exo-miR-103a-3p secreted by CAFs suppressed apoptosis and promoted cisplatin resistance in NSCLC cells.

### Pumilio2 facilitated miR-103a-3p packaging into CAF-derived exosomes

**Figure 3:**
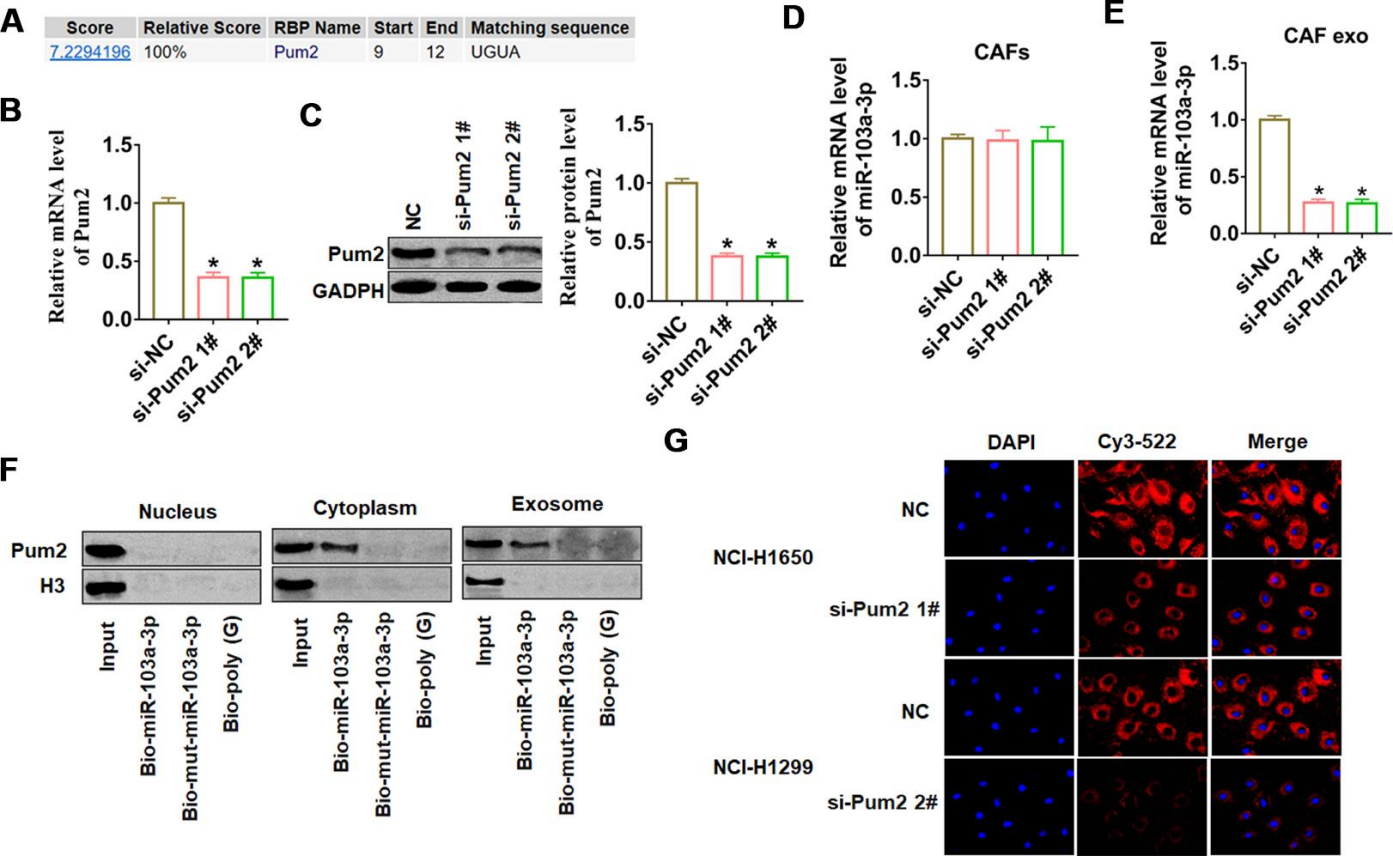
**Pum2 mediated miR-103a-3p packaging into CAF exosomes.** (**A**) RBPDB analysis of the specific interaction between miR-103a-3p and RBP motifs (threshold 0.8). Two siRNAs of Pum2 were transfected into CAFs, and the silencing efficiency was calculated by (**B**) western blot and (**C**) qRT-PCR assay. qRT-PCR assay was used to test the expression of miR-103a-3p in (**D**) CAFs and (**E**) exosomes from CAFs. (**F**) Western blot analysis of Pum2 expression in samples derived by miRNA pulldowns performed with nuclear, cytoplasmic, or exosomal CAFs lysates, biotinylated poly(G) was used as a negative control. (**G**) H1650 and NCI-H1299 cells were co-cultured with CAFs transfected with Cy3-miR-103a-3p and siRNA of Pum2 for 48 h. Fluorescence microscopy was used to detect red fluorescent signals. *p<0.05 vs si-NC.

To investigate whether miR-103a-3p was specifically packaged into exosomes, we used the database of RBP specificities at a threshold of 0.8 (http://rbpdb.ccbr.utoronto.ca/) to search the NA-binding proteins (RBPs) of miR-103a-3p. As shown in Figure 3A, we only predicted that Pumilio2 (Pum2) could bind to miR-103a-3p. Next, we constructed siRNA of Pum2 to silence Pum2 expression in CAFs, and found a remarkable decrease of Pum2 mRNA and protein expression in si-Pum2 transfected CAFs (Figure 3B, 3C). As well, we found si-Pum2 transfection didn’t alter miR-103a-3p expression (Figure 3D), but si-Pum2 inhibited miR-103a-3p expression in CAF exosomes (Figure 3E). Moreover, miRNA pull-down assays showed that Pum2 bound with miR-103a-3p in the cytoplasm and exosomes but not in the nucleus, However, mutant miR-103a-3p with a mutated matching sequence could not bind with Pum2 (Figure 3F). To test the role of Pum2 on miR-103a-3p packaging from CAFs, we co-cultured NCI-H1650 or NCI-H1299 with CAFs transfected with si-Pum2 and Cy3 labeled miR-103a-3p. The fluorescence intensity of Cys-miR-103a-3p was high in NCI-H1650 or NCI-H1299 SGC7901 cells, but si-Pum2 in CAFs blocked the transport of cys-miR-103a-3p from CAFs to NSCLC cells (Figure 3G). These data suggested that Pum2 contributed to miR-103a-3p packaging in CAF-derived exosomes and facilitated miR-103a-3p transport from CAFs to NSCLC cells.

### Pum2 promoted cisplatin resistance via facilitating exo-miR-103a-3p secretion in NSCLC cells

**Figure 4:**
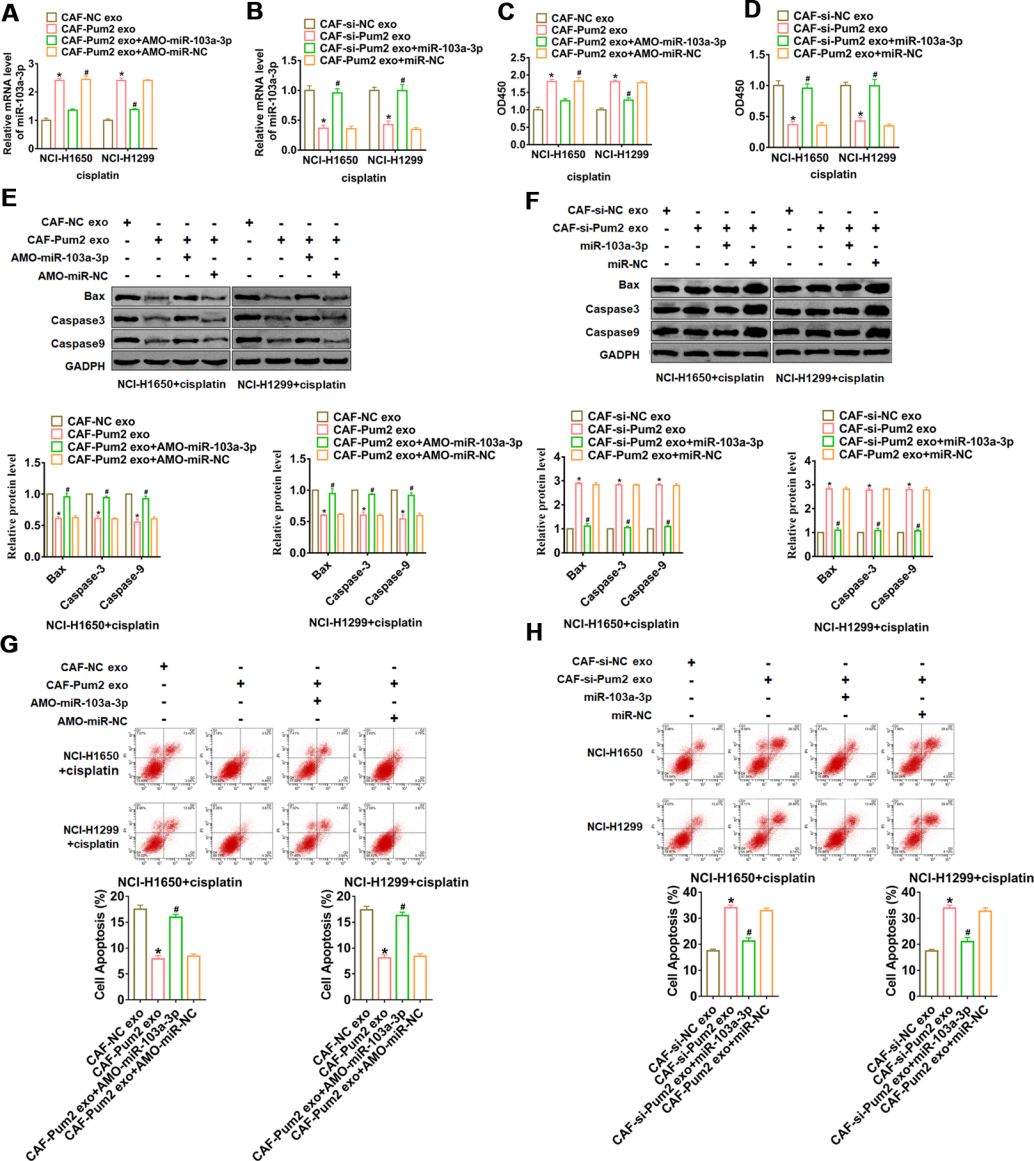
**Pum2 promoted exosomal miR-103a-3p secretion and cisplatin resistance in NSCLC cells.** (**A**) qRT-PCR analyzed the expression of miR-103a-3p NCI-H1650 and NCI-H1299 cells incubated with CAFs transfected Pum2 plasmid with or without AMO-miR-103a-3p. (**B**) qRT-PCR analyzed the expression of miR-103a-3p NCI-H1650 and NCI-H1299 cells incubated with CAFs transfected si-Pum2 with or without miR-103a-3p. (**C**, **D**) CCK8 was used to test viability of NCI-H1650 and NCI-H1299 cells. (**E**, **F**) The expressions of apoptosis related protein Bax, Caspase3 and Caspase9 were analyzed by western bolt. (**G**, **H**) The number of apoptotic cells was calculated by flow cytometry. *p<0.05 vs CAF-NC exo or CAF-si-NC exo, ^#^ p<0.05 vs CAF-Pum2 exo+AMO-miR-103a-3p or CAF-si-Pum2 exo+miR-103a-3p.

The exosomes were isolated from CAFs transfected Pum2 plasmid with or without AMO-miR-103a-3p, si-Pum2 with or without miR-103a-3p, then were added into NCI-H1650 and NCI-H1299 cells. And we found that overexpression of Pum2 promoted miR-103a-3p level in NSCLC cells, while AMO-miR-103a-3p reversed Pum2 effect (Figure 4A). Similarly, si-Pum2 inhibited miR-103a-3p expression, while miR-103a-3p removed si-Pum2 effect (Figure 4B). Followed functional experiments showed that incubation of exosomes from CAFs transfected with Pum2 plasmid promoted cisplatin resistance and suppressed apoptosis in NCI-H1650 and NCI-H1299 cells, while AMO-miR-103a-3p remitted Pum2 function (Figure 4C, 4E, 4G). Analogously, incubation of exosomes from CAFs transfected with si-Pum2 promoted cisplatin sensitivity and apoptosis in NCI-H1650 and NCI-H1299 cells, while AMO-miR-103a-3p relieved si-Pum2 effects (Figure 4D, 4F, 4H).

### Bak1 was a direct target of miR-103a-3p

**Figure 5:**
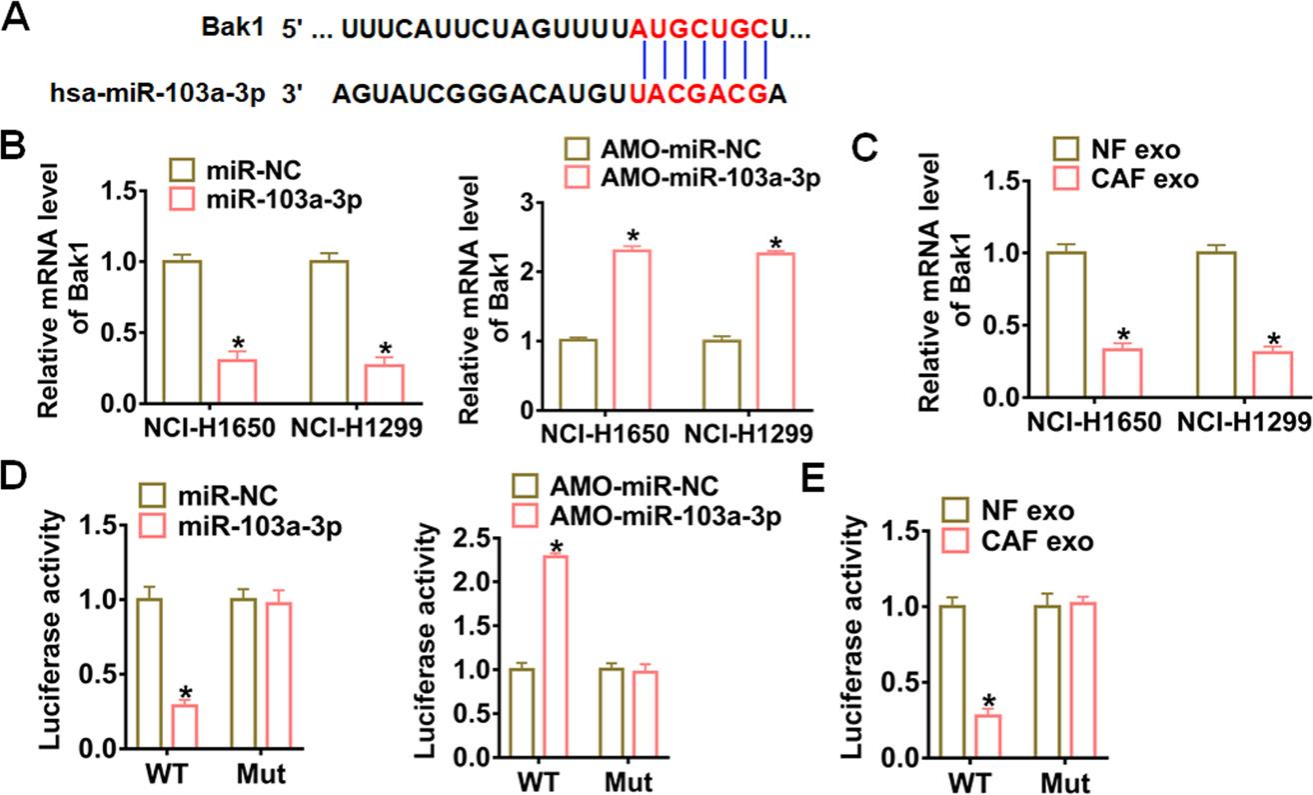
**Bak1 was a direct target of exosomal miR-103a-3p in NSCLC cells.** (**A**) Predicted miR-103a-3p target sequences in the 3′ UTRs of Bak1 genes. (**B**) Bak1 mRNA expression in NCI-H1650 and NCI-H1299 transfected with miR-103a-3p or AMO- miR-103a-3p at 48 h after transfection. (**C**) Bak1 mRNA levels in NCI-H1650 and NCI-H1299 cells at 48 h after incubation with CAF exosomes. (**D**) WT and mutant Bak1 luciferase plasmids were transfected into HEK293 cells with miR-103a-3p or AMO- miR-103a-3p. The luciferase activity was measured by dual-luciferase reporter assay system. (**E**) The effects of CAF exosomes on Bak1 reporter luciferase activity in HEK293 cells. *p<0.05 vs miR-NC or AMO-miR-NC or NF exo.

Targetscan prediction data showed that the 3’UTR of Bak1 possessed paired bases for miR-103a-3p (Figure 5A). qRT-PCR analysis revealed that miR-103a-3p significantly inhibited Bak1 expression in NCI-H1650 and NCI-H1299 cells, while AMO-miR-103a-3p increased Bak1 expression (Figure 5B). What’s more, CAF exosomes obviously suppressed Bak1 expression in NCI-H1650 and NCI-H1299 cells (Figure 5C). To investigate whether miR-103a-3p targeted on Bak1, we carried out dual-luciferase reporter assay in HEK293 cell line. miR-103a-3p inhibited the luciferase activity of WT Bak1, but not the mutant Bak1 (Figure 5D). Similarly, AMO-miR-103a-3p promoted the luciferase activity of WT of Bak1 rather than mutant of Bak1 (Figure 5D). As expect, CAF exosomes also effectively downregulated the WT Bak1 luciferase activity of (Figure 5E).

### Exo-miR-103a-3p accelerated cisplatin resistance in NSCLC cells via Bak1 downregulation

**Figure 6:**
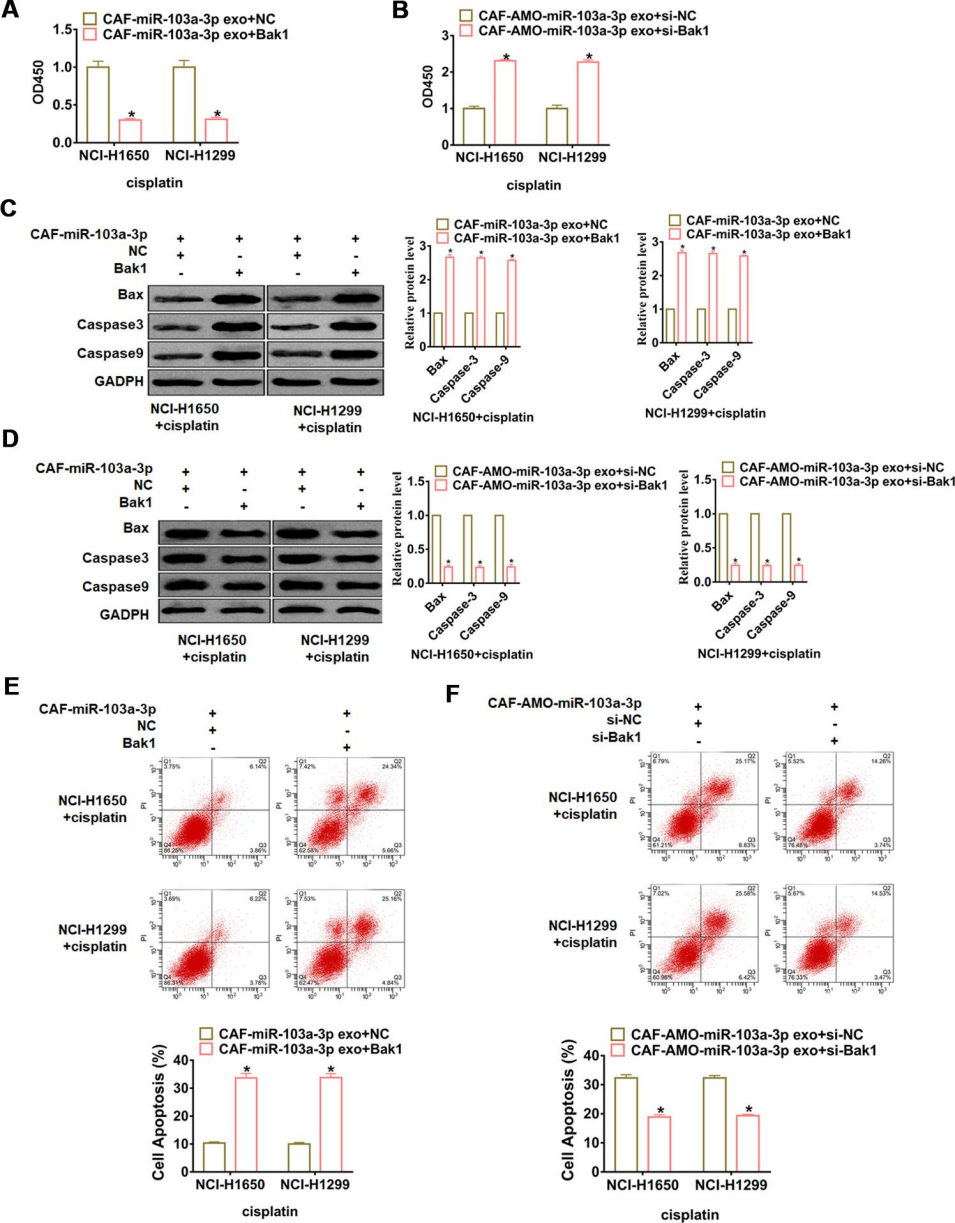
**miR-103a-3p promotes cisplatin resistance in NSCLC cells by inhibiting Bak1.** NCI-H1650 and NCI-H1299 cells were transfected with Bak1 or si- Bak1 and incubated with exosomes from CAFs transfected with miR-103a-3p or AMO-103a-3p, respectively. (**A**, **B**) CCK8 was used to test viability of NCI-H1650 and NCI-H1299 cells. (**C**, **D**) The expressions of apoptosis related protein Bax, Caspase3 and Caspase9 were analyzed by western bolt. (**E**, **F**) The number of apoptotic cells was calculated by flow cytometry. *p<0.05 vs CAF-miR-103a-3p exo+NC or CAF-AMO-miR-103a-3p exo+si-NC.

To further elucidate the functional role of the Bak1 in miR-103a-3p promoting cisplatin resistance, NCI-H1650 and NCI-H1299 cells were transfected with Bak1 or si- Bak1 and incubated with exosomes from CAFs transfected with miR-103a-3p or AMO-103a-3p, respectively. Followed functional experiments showed that overexpression of Bak1 promoted cisplatin sensitivity and apoptosis in NCI-H1650 and NCI-H1299 cells (Figure 6A, 6C, 6E), while silencing of Bak1 repressed cisplatin sensitivity and apoptosis in NCI-H1650 and NCI-H1299 cells (Figure 6B, 6D, 6F).

### CAF-derived exosomal miR-103a-3p promoted NSCLC cisplatin resistance by suppressing apoptosis *in vivo*

**Figure 7:**
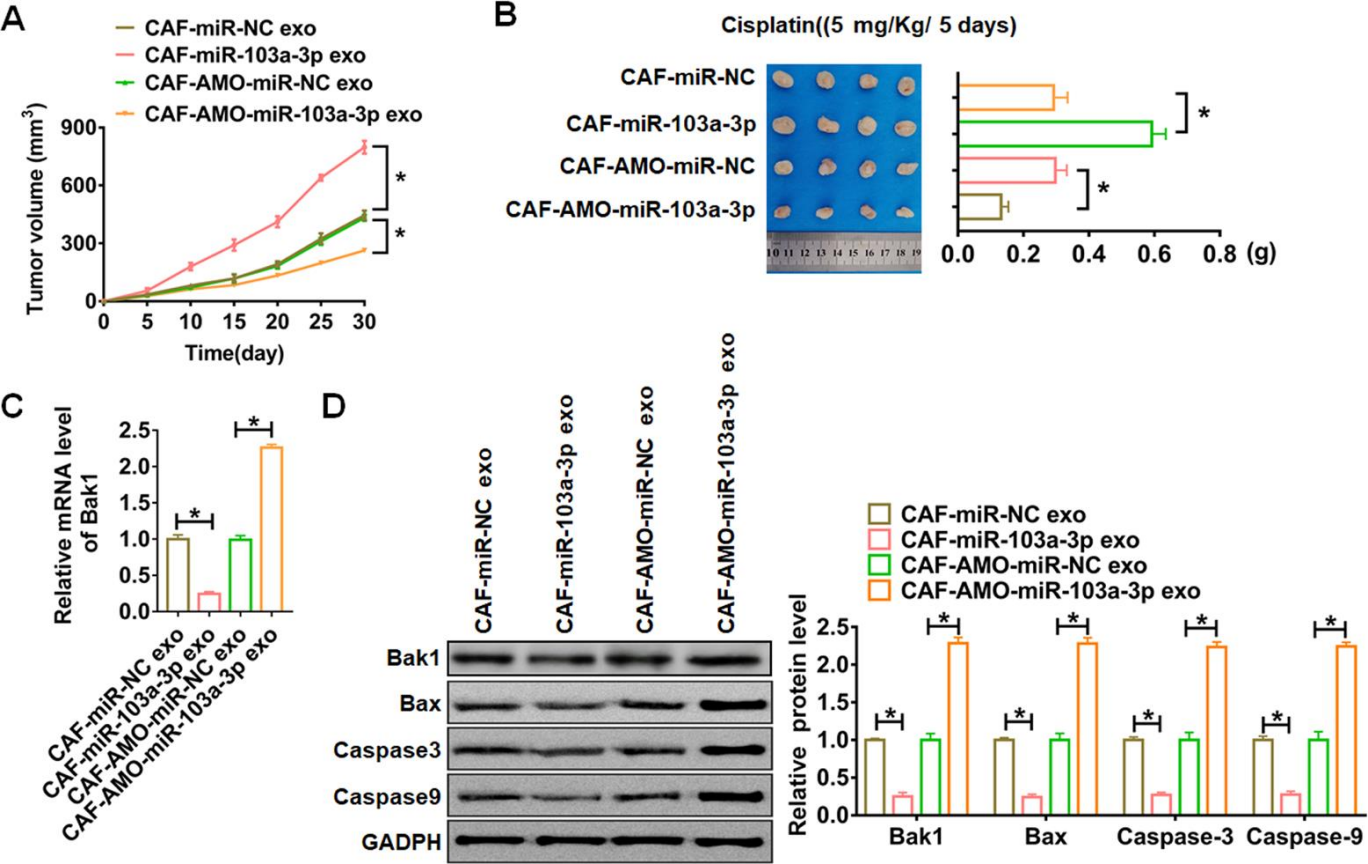
***In vivo* role of exo-miR-103a-3p in regulating apoptosis and chemo-sensitivity of NSCLC tumors.** The nude mice were injected NCI-H1650 cells transfected with or without a mixture of CAFs transfected with miR-103a-3p or AMO-miR-103a-3p or NC. These mice were then injected with either cisplatin (5 mg/Kg/ 5 days) or saline after 7days of cells injection. (**A**) Alterations of tumor diameters in each group. (**B**) Weight measurements of the tumors. (**C**) qRT-PCR analyzed Bak1 mRNA levels in tumors. (**D**) The protein expression of Bak1 and apoptosis related protein Bax, Caspase3 and Caspase9 were analyzed by western bolt. *p<0.05.

Finally, the nude mice were injected NCI-H1650 cells transfected with or without a mixture of CAFs transfected with miR-103a-3p or AMO-miR-103a-3p or NC, then intraperitoneally injected with either cisplatin (5 mg/Kg/ 5 days) or saline after 7days of cells injection. Tumors grew faster and bigger in the mice with CAFs-miR-103a-3p, while CAFs-AMO-miR-103a-3p inhibited the growth rate and volume of tumors (Figure 7A).

**Figure 8:**
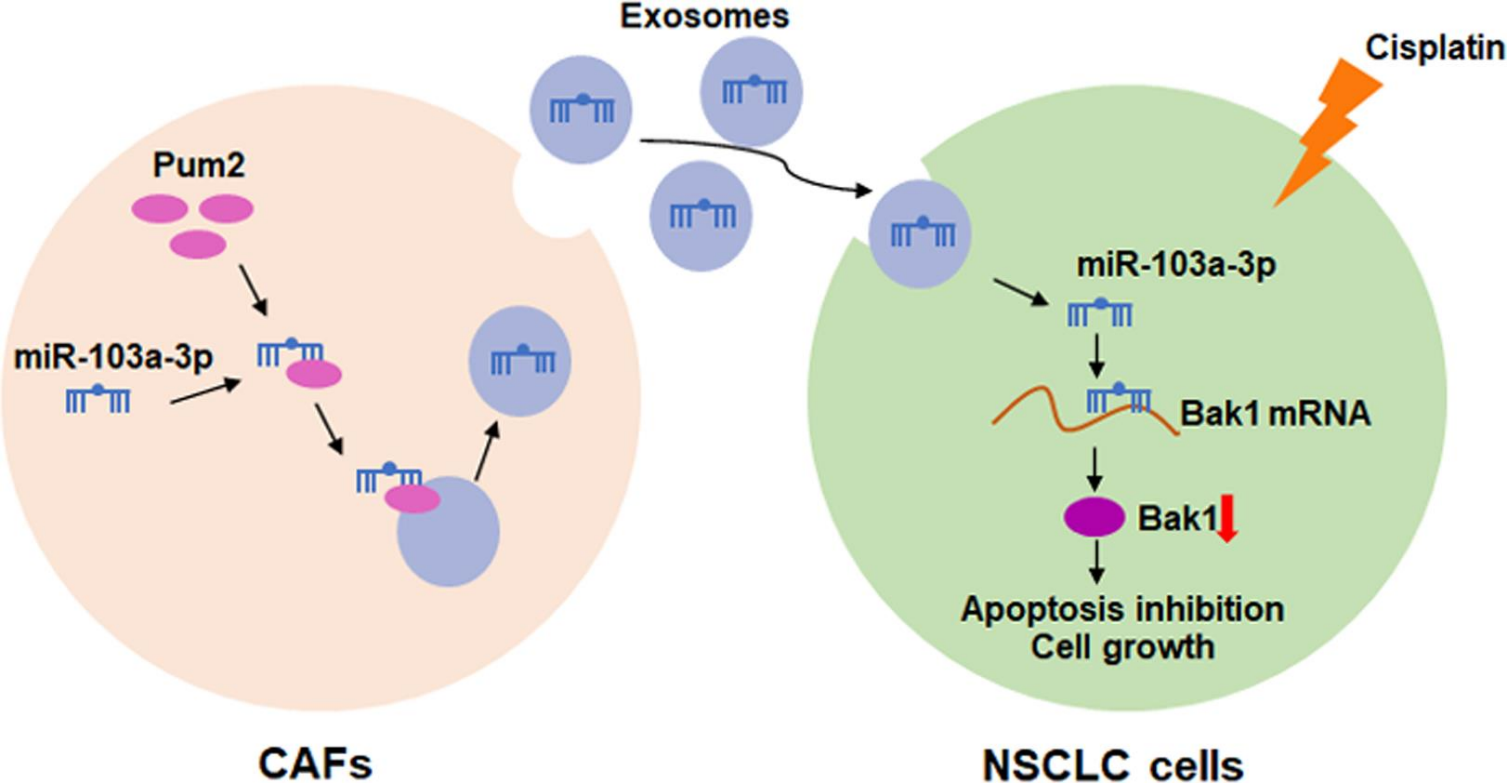
Hypothesis diagram illustrates function and mechanism of exosomal miR-103a-3p from CAFs in NSCLC progress.

The tumors were isolated at 30 days after injection, CAFs-miR-103a-3p significantly increased tumors weight, and CAFs-AMO-miR-103a-3p decreased tumors weight (Figure 7B). In addition, miR-103a-3p decreased the mRNA expression of Bak1 in tumor tissues, while AMO-miR-103a-3p showed the opposite effect (Figure 7C). Moreover, miR-103a-3p decreased the protein expression of Bak1 and apoptosis related genes, but AMO-miR-103a-3p played the opposite role (Figure 7D, Figure 8).

## DISCUSSION

NSCLC has the highest incidence rate of lung cancer [13], which mainly treated with cisplatin-containing regimens in clinical chemotherapy [14]. However, the therapeutic effect of these regimens is not satisfactory, mainly due to cisplatin resistance [15]. At present, there is no new drug that can effectively reverse the cisplatin resistance of NSCLC, and it has become a focus to alleviate cisplatin resistance in NSCLC process.

MiRNA is a kind of endogenous non-coding small molecule RNA, which is 19 to 24 nucleotides long and exists widely in eukaryotes [16]. Studies have shown that miRNA and its mediated signaling pathways are directly involved in the regulation of multiple cellular biological pathways and cisplatin responses in NSCLC [17]. It was showed that overexpression of miR-34a in A549 cells increased the sensitivity to cisplatin and decreased drug resistance-related proteins significantly [18]. The proliferation, invasion and metastasis of cancer is a long-term and complex process involving multiple factors and mechanisms [19]. The formation of NSCLC microenvironment can promote the rapid growth of lung cancer cells and increase the invasion and metastasis ability of tumor cells [20]. It was showed that exosomes from the supernatant of A549 cell culture of lung cancer increased cancer progression and decreased apoptosis level [21]. In present study, we isolated CAFs and CAF exosomes from NSCLC tissues. Interestingly, exosomal miR-103a-3p derived from CAFs in NSCLC. Our data indicated CAFs secreted exosomes containing microRNA in NSCLC, which was similar with other studies. It has been shown that CAFs could secret exo-miR-522 in gastric cancer [22].

Apoptosis usually occurs during normal cell development and maintains homeostasis [23]. Abnormal apoptosis can activate proto-oncogenes and promote the survival of tumor cells [24]. The tumor suppressor gene p53 is a regulatory factor of cell apoptosis [25]. TP53 mutation or deletion can promote the formation of various tumors [26]. Considering the treatment status of NSCLC and the important role of apoptosis in tumor development, we wondered exo-miR-103a-3p effects in cisplatin resistance and apoptosis of NSCLC. We isolated CAFs exosomes and co-cultured with NCI-H1650 and NCI-H1299 cells, and found that CAF exosomes entered NCI-H1650 and NCI-H1299 cells and promoted the expression of exo-miR-103a-3p. Notably, exo-miR-103a-3p derived from CAFs promoted cisplatin resistance and inhibited apoptosis in NSCLC cells.

Exosome transport is an effective way to regulate signal transduction and biological functions of receptor cells [27]. And Exosome packaging microRNAs requires the involvement of regulatory factors, hnRNPA1 accelerated exosomal miR-196a packaging in head and neck cancer [28]. In present study, we found that RNA binding protein Pum2 bound with miR-103a-3p and facilitated its packaging into CAF-derived exosomes in NSCLC cells. Pum2 has been showed to play a key role in cancer development. LncRNA TUG1 bound with Pum2 and then promoted cervical cancer progression [29]. As well, Pum2 regulated the stemness of breast cancer cells via binding with miR-376a [30]. Further exploring showed the pro-apoptosis gene Bak1 was a direct target of miR-103a-3p, and miR-103a-3p accelerated cisplatin resistance in NSCLC cells via Bak1 downregulation. *In vivo* tumorigenesis assay showed CAF-derived exosomal miR-103a-3p enhanced cisplatin resistance and inhibited cell apoptosis in NSCLC.

It has been proved that miRNA is involved in the occurrence and development of cancers, cisplatin resistance of chemotherapy drugs and the sensitivity of patients to radiotherapy and gene therapy. It indicates that miRNA can improve the current treatment status of NSCLC patients and bring good news for their treatment and prognosis. Unfortunately, the current studies on miRNA regulation of cisplatin resistance in NSCLC are mostly limited to the candidate target level, and there is a lack of exploration on elucidating the cisplatin resistance mechanism at the miRNA expression network level. It is hoped that further research and exploration are needed.

## MATERIALS AND METHODS

### Clinical samples

Fresh cancer tissue samples and para-carcinoma tissue samples were taken from 15 NSCLC patients undergoing surgical procedures at the First Affiliated Hospital of Xinxiang Medical University. All of the patients or their guardians provided written consent, and the Ethics Committee of the First Affiliated Hospital of Xinxiang Medical University.

### CAFs and exosome isolation

CAFs and NFs were isolated from cancer and para-carcinoma tissues of NSCLC patients, which were identified by specific makers α-SMA, FAP and FSP1. We collected the culture medium after 48 hours. Several centrifugations were performed to purify exosomes. Briefly, we centrifuged the supernatant at 300 g, 2,000 g and 10,000 g for 10 minutes, respectively. And then, supernatant was filtrated and purified by ultracentrifugation at 100,000 g. CAFs-derived exosomes were analyzed using exosome marker protein CD63, Tsg101 and ALIX via Western blot.

### Transmission electron microscopy (TEM)

TEM was used to identify exosomes structures. Exosomes were stained by by 2% phosphotungstic acid for 5 min, and then observed by transmission electron microscope.

### Cell culture and treatment

The NCI-H1650 and NCI-H1299 lines were purchased from the Science Cell Laboratory. Cells were cultured in PRIM 1640 (GIBCO, USA) supplemented with 10 % fetal bovine serum (Cromwell, USA) and 100 μL/mL penicillin and streptomycin (Sigma-Aldrich, USA) and placed at 37° C with 5% CO2. 2 μg plasmid or 500 nM si-RNA/microRNA/antisense morpholino oligonucleotide (AMO)-microRNA or its NC was transfected into cells with LipofectamineTM 2000 (Invitrogen, Carlsbad, CA, USA), respectively. 10 μM cisplatin was added into medium for cisplatin treatment. As for co-culture assay, we used transwell membranes in 12-well plates. CAFs or NFs were plated into the upper chamber, and NCI-H1650 and NCI-H1299 was cultured in lower chamber.

### qRT-PCR

RNA isolation, reverse transcription and quantitative expression were carried according to manufacturer’s instructions. All the kits were purchased from Vazyme, and gene expression was calculated using 2-ΔΔCt method.

### Protein isolation and western blot

Total protein was collected from cells with RIPA lysis Mix. Western blotting assay was performed as previously described [12]. Briefly, 60 μg protein extractions were loaded via SDS-PAGE and transferred onto nitrocellulose membranes (absin, China), then incubated with primary antibodies for 2 hrs at temperature, then plated at 4° C overnight, the membranes were incubated in 5% non-fat milk blocking buffer for horizontal mode 3 h. After incubation with secondary antibodies, the membranes were scanned using an Odyssey, and data were analyzed with Odyssey software (LI-COR, USA).

### Immunofluorescence staining

Cells were plated in a 24-well cell culture plate. After transfection, cells were washed by PBS and fixed with 4% paraformaldehyde. Cells were permeabilized with 0.2% Triton-X-100 solution in PBS. Next, we blocked cell using goat serum. Then, the cells were incubated antibody at 4° C overnight followed with FITC-conjugated goat anti-mouse antibodies incubation for 1h. After three washes with PBS, we incubated cells by DAPI.

### Biotin miRNA pull-down assay

The solution of exosomes was incubated with miR-103a-3p containing a biotin modification overnight. And agarose beads were added into the solution and mixed for 4 h. The precipitates were separated and detected by western blotting analysis.

### Luciferase assay

psiCHECK-2 luciferase reporter plasmid was inserted with the wildtype Bak1 3’UTR or mutant 3’UTR sequences, then were transfected with reporter vectors into HEK293 cells. The cells were collected after 48 h post-transfection and lysed to detect the luciferase activity (Promega, USA).

### CCK8 assay

Cells were seeded in 96-well cell plates, and added CCK-8 solution (Vazyme, China) at 48 h. 2 hours later, measure the OD value at 450 nm.

### *In vivo* tumor growth assay

Nude mice were purchased from Guangdong provincial experimental animal center for medicine. The nude mice were injected NCI-H1650 cells transfected with or without a mixture of CAFs transfected with miR-103a-3p or AMO-miR-103a-3p or NC. cisplatin (5 mg/kg/ 5 days) or saline were intraperitoneally injected into mice after 7days of cells injection. Tumor size was measured every five days. After 30 days of injection, mice were intraperitoneally injected with 3% pentobarbital sodium and were killed by excessive anesthesia with a dose of 90 mL/kg, and the tumors were removed for follow-up study.

### Cell apoptosis assay

Cell apoptosis was calculated by Annexin V apoptosis kit (Beyotime, China), and the operating procedure was according to the kit instructions. Briefly, 5×10^5^ cells/mL were centrifuged and resuspended in with Annexin V-FITC and PI solution in darkness for 15 min. Then, Binding Buffer was mixed into the resuspension and detected with instrument. Cell apoptosis level was detected within 1 h.

### Statistical analysis

Significant differences were calculated using two-tailed t-test through Graphpad 7.0 and SPSS 22.0.

## References

[1] Piotrowska Z, Costa DB, Oxnard GR, Huberman M, Gainor JF, Lennes IT, Muzikansky A, Shaw AT, Azzoli CG, Heist RS, Sequist LV. Activity of the Hsp90 inhibitor luminespib among non-small-cell lung cancers harboring EGFR exon 20 insertions. Ann Oncol. 2018; 29:2092–97. 10.1093/annonc/mdy336 30351341

[2] Chen W, Zheng R, Baade PD, Zhang S, Zeng H, Bray F, Jemal A, Yu XQ, He J. Cancer statistics in China, 2015. CA Cancer J Clin. 2016; 66:115–32. 10.3322/caac.21338 26808342

[3] Ettinger DS, Wood DE, Akerley W, Bazhenova LA, Borghaei H, Camidge DR, Cheney RT, Chirieac LR, D’Amico TA, Dilling TJ, Dobelbower MC, Govindan R, Hennon M, et al. NCCN guidelines insights: non-small cell lung cancer, version 4.2016. J Natl Compr Canc Netw. 2016; 14:255–64. 10.6004/jnccn.2016.0031 26957612 PMC10181272

[4] Zhou F, Jiang T, Ma W, Gao G, Chen X, Zhou C. The impact of clinical characteristics on outcomes from maintenance therapy in non-small cell lung cancer: a systematic review with meta-analysis. Lung Cancer. 2015; 89:203–11. 10.1016/j.lungcan.2015.06.005 26115839

[5] Lin YX, Wang Y, An HW, Qi B, Wang J, Wang L, Shi J, Mei L, Wang H. Peptide-based autophagic gene and cisplatin co-delivery systems enable improved chemotherapy resistance. Nano Lett. 2019; 19:2968–78. 10.1021/acs.nanolett.9b00083 30924343

[6] Yokoi A, Villar-Prados A, Oliphint PA, Zhang J, Song X, De Hoff P, Morey R, Liu J, Roszik J, Clise-Dwyer K, Burks JK, O’Halloran TJ, Laurent LC, Sood AK. Mechanisms of nuclear content loading to exosomes. Sci Adv. 2019; 5:eaax8849. 10.1126/sciadv.aax8849 31799396 PMC6867874

[7] Wang Y, Yi J, Chen X, Zhang Y, Xu M, Yang Z. The regulation of cancer cell migration by lung cancer cell-derived exosomes through TGF-β and IL-10. Oncol Lett. 2016; 11:1527–30. 10.3892/ol.2015.4044 26893774 PMC4734314

[8] Liang Y, Zhang D, Li L, Xin T, Zhao Y, Ma R, Du J. Exosomal microRNA-144 from bone marrow-derived mesenchymal stem cells inhibits the progression of non-small cell lung cancer by targeting CCNE1 and CCNE2. Stem Cell Res Ther. 2020; 11:87. 10.1186/s13287-020-1580-7 32102682 PMC7045474

[9] Hu JL, Wang W, Lan XL, Zeng ZC, Liang YS, Yan YR, Song FY, Wang FF, Zhu XH, Liao WJ, Liao WT, Ding YQ, Liang L. CAFs secreted exosomes promote metastasis and chemotherapy resistance by enhancing cell stemness and epithelial-mesenchymal transition in colorectal cancer. Mol Cancer. 2019; 18:91. 10.1186/s12943-019-1019-x 31064356 PMC6503554

[10] Apollonio B, Ramsay AG. Exosomes and CAFs: partners in crime. Blood. 2015; 126:1053–55. 10.1182/blood-2015-07-655233 26316614

[11] Wang H, Wei H, Wang J, Li L, Chen A, Li Z. MicroRNA-181d-5p-containing exosomes derived from CAFs promote EMT by regulating CDX2/HOXA5 in breast cancer. Mol Ther Nucleic Acids. 2020; 19:654–67. 10.1016/j.omtn.2019.11.024 31955007 PMC6970169

[12] Guo H, Ha C, Dong H, Yang Z, Ma Y, Ding Y. Cancer-associated fibroblast-derived exosomal microRNA-98-5p promotes cisplatin resistance in ovarian cancer by targeting CDKN1A. Cancer Cell Int. 2019; 19:347. 10.1186/s12935-019-1051-3 31889899 PMC6925473

[13] Ni M, Liu X, Wu J, Zhang D, Tian J, Wang T, Liu S, Meng Z, Wang K, Duan X, Zhou W, Zhang X. Identification of candidate biomarkers correlated with the pathogenesis and prognosis of non-small cell lung cancer via integrated bioinformatics analysis. Front Genet. 2018; 9:469. 10.3389/fgene.2018.00469 30369945 PMC6194157

[14] Rinaldi M, Cauchi C, Gridelli C. First line chemotherapy in advanced or metastatic NSCLC. Ann Oncol. 2006 (Suppl 5); 17:v64–67. 10.1093/annonc/mdj953 16807466

[15] Moro M, Caiola E, Ganzinelli M, Zulato E, Rulli E, Marabese M, Centonze G, Busico A, Pastorino U, de Braud FG, Vernieri C, Simbolo M, Bria E, et al. Metformin enhances cisplatin-induced apoptosis and prevents resistance to cisplatin in co-mutated KRAS/LKB1 NSCLC. J Thorac Oncol. 2018; 13:1692–704. 10.1016/j.jtho.2018.07.102 30149143

[16] Gutbrod MJ, Martienssen RA. Conserved chromosomal functions of RNA interference. Nat Rev Genet. 2020; 21:311–31. 10.1038/s41576-019-0203-6 32051563 PMC9478574

[17] Xue W, Dahlman JE, Tammela T, Khan OF, Sood S, Dave A, Cai W, Chirino LM, Yang GR, Bronson R, Crowley DG, Sahay G, Schroeder A, et al. Small RNA combination therapy for lung cancer. Proc Natl Acad Sci USA. 2014; 111:E3553–61. 10.1073/pnas.1412686111 25114235 PMC4151750

[18] Jiao P, Hou J, Yao M, Wu J, Ren G. SNHG14 silencing suppresses the progression and promotes cisplatin sensitivity in non-small cell lung cancer. Biomed Pharmacother. 2019; 117:109164. 10.1016/j.biopha.2019.109164 31252267

[19] Liu F, Cai Y, Rong X, Chen J, Zheng D, Chen L, Zhang J, Luo R, Zhao P, Ruan J. MiR-661 promotes tumor invasion and metastasis by directly inhibiting RB1 in non small cell lung cancer. Mol Cancer. 2017; 16:122. 10.1186/s12943-017-0698-4 28716024 PMC5514511

[20] Chen YL, Zhang Y, Wang J, Chen N, Fang W, Zhong J, Liu Y, Qin R, Yu X, Sun Z, Gao F. A 17 gene panel for non-small-cell lung cancer prognosis identified through integrative epigenomic-transcriptomic analyses of hypoxia-induced epithelial-mesenchymal transition. Mol Oncol. 2019; 13:1490–502. 10.1002/1878-0261.12491 30973670 PMC6599842

[21] Li H, Zhang Q, Wu Q, Cui Y, Zhu H, Fang M, Zhou X, Sun Z, Yu J. Interleukin-22 secreted by cancer-associated fibroblasts regulates the proliferation and metastasis of lung cancer cells via the PI3K-Akt-mTOR signaling pathway. Am J Transl Res. 2019; 11:4077–88. 31396319 PMC6684901

[22] Zhang H, Deng T, Liu R, Ning T, Yang H, Liu D, Zhang Q, Lin D, Ge S, Bai M, Wang X, Zhang L, Li H, et al. CAF secreted miR-522 suppresses ferroptosis and promotes acquired chemo-resistance in gastric cancer. Mol Cancer. 2020; 19:43. 10.1186/s12943-020-01168-8 32106859 PMC7045485

[23] Walensky LD. Targeting BAX to drug death directly. Nat Chem Biol. 2019; 15:657–65. 10.1038/s41589-019-0306-6 31209350

[24] Ma G, Liu H, Hua Q, Wang M, Du M, Lin Y, Ge Y, Gong W, Zhao Q, Qiang F, Tao G, Zhang Z, Chu H. KCNMA1 cooperating with PTK2 is a novel tumor suppressor in gastric cancer and is associated with disease outcome. Mol Cancer. 2017; 16:46. 10.1186/s12943-017-0613-z 28231797 PMC5324255

[25] Liu P, Fu W, Verwilst P, Won M, Shin J, Cai Z, Tong B, Shi J, Dong Y, Kim JS. MDM2-Associated Clusterization-Triggered Emission and Apoptosis Induction Effectuated by a Theranostic Spiropolymer. Angew Chem Int Ed Engl. 2020; 59:8435–39. 10.1002/anie.201916524 32052897

[26] Mukhopadhyay UK, Oturkar CC, Adams C, Wickramasekera N, Bansal S, Medisetty R, Miller A, Swetzig WM, Silwal-Pandit L, Børresen-Dale AL, Creighton CJ, Park JH, Konduri SD, et al. TP53 status as a determinant of pro- vs anti-tumorigenic effects of estrogen receptor-beta in breast cancer. J Natl Cancer Inst. 2019; 111:1202–15. 10.1093/jnci/djz051 30990221 PMC6855950

[27] Zhang H, Freitas D, Kim HS, Fabijanic K, Li Z, Chen H, Mark MT, Molina H, Martin AB, Bojmar L, Fang J, Rampersaud S, Hoshino A, et al. Identification of distinct nanoparticles and subsets of extracellular vesicles by asymmetric flow field-flow fractionation. Nat Cell Biol. 2018; 20:332–43. 10.1038/s41556-018-0040-4 29459780 PMC5931706

[28] Qin X, Guo H, Wang X, Zhu X, Yan M, Wang X, Xu Q, Shi J, Lu E, Chen W, Zhang J. Exosomal miR-196a derived from cancer-associated fibroblasts confers cisplatin resistance in head and neck cancer through targeting CDKN1B and ING5. Genome Biol. 2019; 20:12. 10.1186/s13059-018-1604-0 30642385 PMC6332863

[29] Duan W, Nian L, Qiao J, Liu NN. LncRNA TUG1 aggravates the progression of cervical cancer by binding PUM2. Eur Rev Med Pharmacol Sci. 2019; 23:8211–18. 10.26355/eurrev_201910_19128 31646551

[30] Zhang L, Chen Y, Li C, Liu J, Ren H, Li L, Zheng X, Wang H, Han Z. RNA binding protein PUM2 promotes the stemness of breast cancer cells via competitively binding to neuropilin-1 (NRP-1) mRNA with miR-376a. Biomed Pharmacother. 2019; 114:108772. 10.1016/j.biopha.2019.108772 30909144

